# Supplementary figures and images for: Loss of SDHB Induces a Metabolic Switch in the hPheo1 Cell Line toward Enhanced OXPHOS
Source: Int J Mol Sci. 2022 Jan 5;23(1):560. doi: 10.3390/ijms23010560 (PMC8745660; doi:10.3390/ijms23010560)

# 20 nucleotides deletion

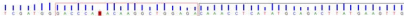

Wild-type SDHB sequence

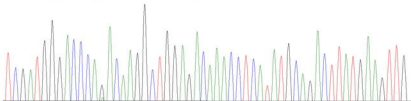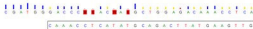

Mutated SDHB sequence

c.142\_161del

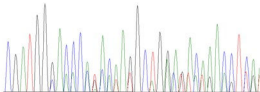

Supplement: Supplementary file 1 [file ijms-23-00560-s001.zip › Figure S1.pdf]

**A**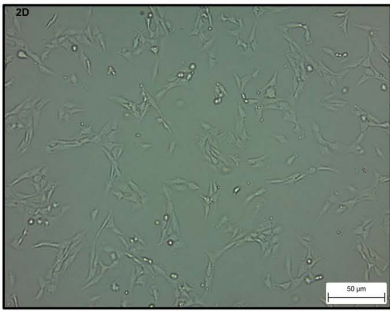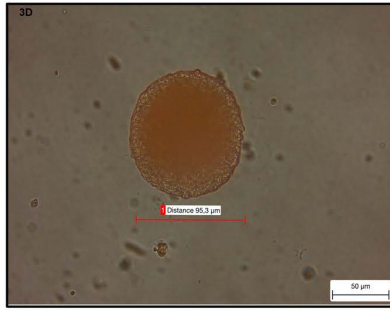**B**

Hallmark gene sets "3D culture"

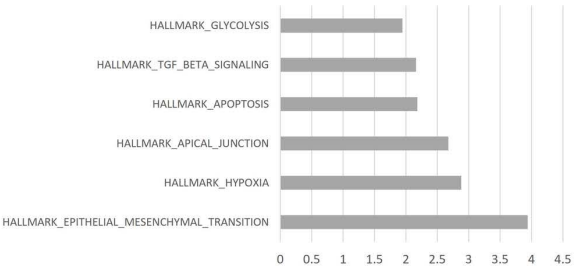

Canonical pathways "3D culture"

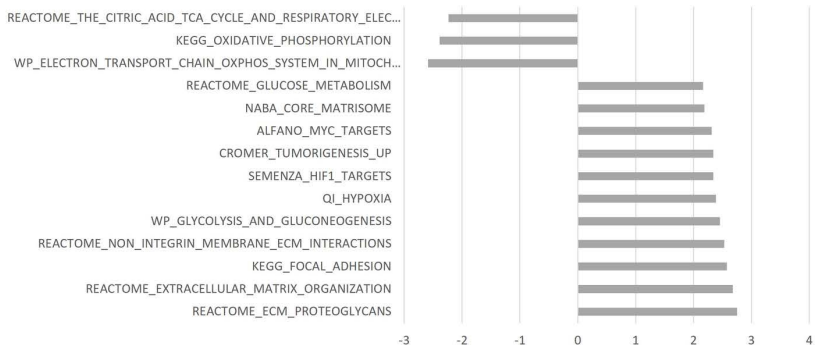

Supplement: Supplementary file 1 [file ijms-23-00560-s001.zip › Figure S2.pdf]

***hPheo1***

***KD-SDHB***

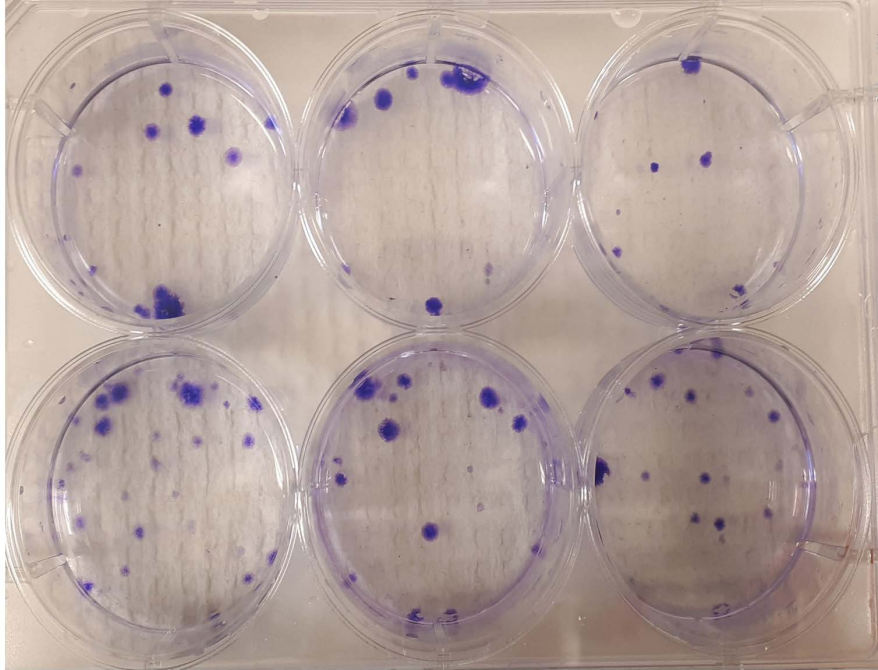

Seeding density= 100 cells/well

Duration: 2 weeks

Survival fraction= 2.78%

Supplement: Supplementary file 1 [file ijms-23-00560-s001.zip › Figure S3.pdf]

A

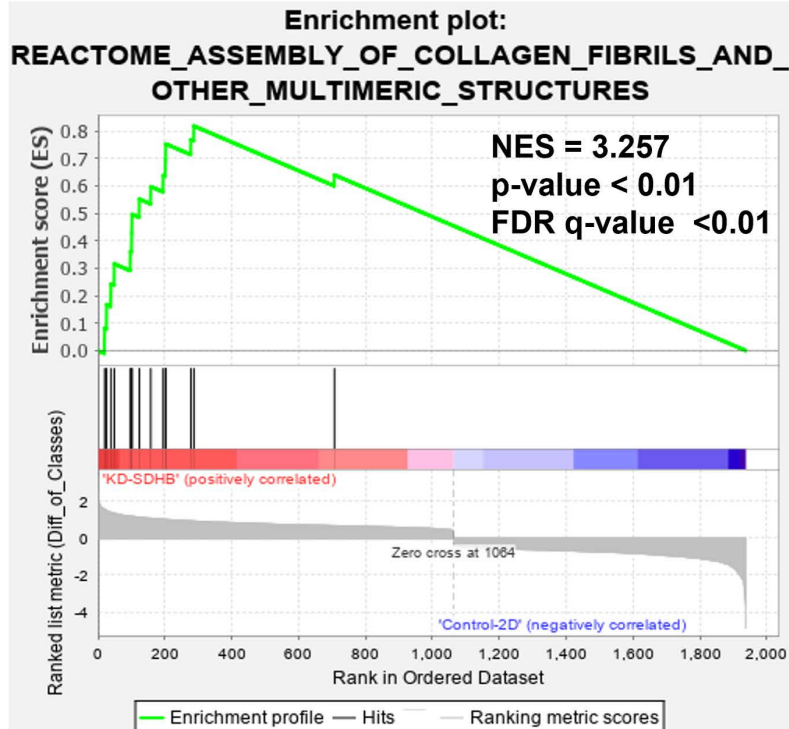

B

KD-SDHB hPeo1

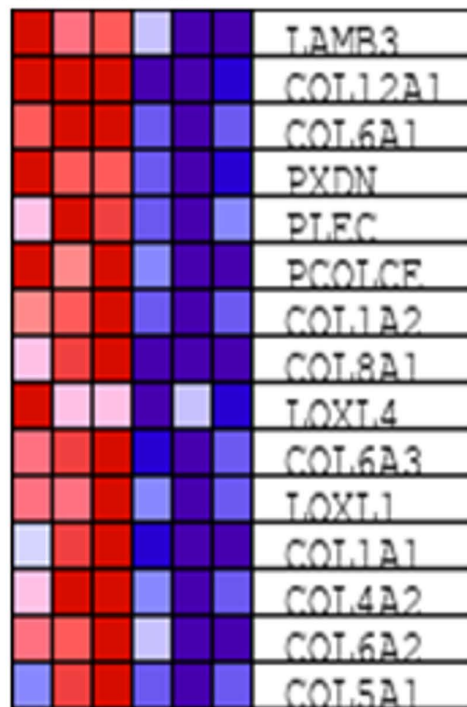

Supplement: Supplementary file 1 [file ijms-23-00560-s001.zip › Figure S4.pdf]

# GLUD1

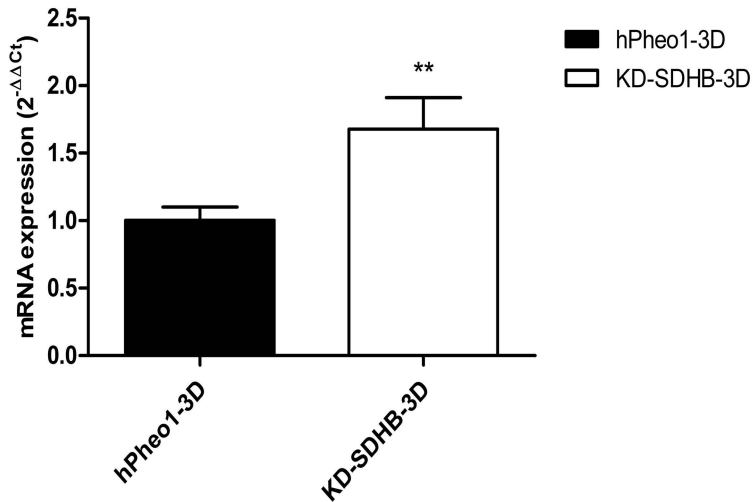

Supplement: Supplementary file 1 [file ijms-23-00560-s001.zip › Figure S5.pdf]
